# Development of a fully automated surgical site infection detection algorithm for use in cardiac and orthopedic surgery research

**DOI:** 10.1017/ice.2020.1387

**Published:** 2021-10

**Authors:** Hiroyuki Suzuki, Gosia S. Clore, Eli N. Perencevich, Stacey M. Hockett-Sherlock, Michihiko Goto, Rajeshwari Nair, Westyn Branch-Elliman, Kelly K. Richardson, Kalpana Gupta, Brice F. Beck, Bruce Alexander, Erin C. Balkenende, Marin L. Schweizer

**Affiliations:** 1 Center for Access & Delivery Research & Evaluation (CADRE), Iowa City Veterans’ Affairs Health Care System, Iowa City, Iowa; 2 Department of Internal Medicine, University of Iowa Carver College of Medicine, Iowa City, Iowa; 3 Division of Infectious Diseases, Department of Medicine, Boston VA Healthcare System, West Roxbury, Massachusetts; 4 Center for Healthcare Organization and Implementation Research (CHOIR), Boston VA Healthcare System, Boston, Massachusetts; 5 Harvard Medical School, Boston, Massachusetts; 6 Boston University School of Medicine, Boston, Massachusetts

## Abstract

**Objective:**

To develop a fully automated algorithm using data from the Veterans’ Affairs (VA) electrical medical record (EMR) to identify deep-incisional surgical site infections (SSIs) after cardiac surgeries and total joint arthroplasties (TJAs) to be used for research studies.

**Design:**

Retrospective cohort study.

**Setting:**

This study was conducted in 11 VA hospitals.

**Participants:**

Patients who underwent coronary artery bypass grafting or valve replacement between January 1, 2010, and March 31, 2018 (cardiac cohort) and patients who underwent total hip arthroplasty or total knee arthroplasty between January 1, 2007, and March 31, 2018 (TJA cohort).

**Methods:**

Relevant clinical information and administrative code data were extracted from the EMR. The outcomes of interest were mediastinitis, endocarditis, or deep-incisional or organ-space SSI within 30 days after surgery. Multiple logistic regression analysis with a repeated regular bootstrap procedure was used to select variables and to assign points in the models. Sensitivities, specificities, positive predictive values (PPVs) and negative predictive values were calculated with comparison to outcomes collected by the Veterans’ Affairs Surgical Quality Improvement Program (VASQIP).

**Results:**

Overall, 49 (0.5%) of the 13,341 cardiac surgeries were classified as mediastinitis or endocarditis, and 83 (0.6%) of the 12,992 TJAs were classified as deep-incisional or organ-space SSIs. With at least 60% sensitivity, the PPVs of the SSI detection algorithms after cardiac surgeries and TJAs were 52.5% and 62.0%, respectively.

**Conclusions:**

Considering the low prevalence rate of SSIs, our algorithms were successful in identifying a majority of patients with a true SSI while simultaneously reducing false-positive cases. As a next step, validation of these algorithms in different hospital systems with EMR will be needed.

Studies of interventions to decrease the incidence of surgical site infections (SSIs) must include thousands of patients to achieve sufficient statistical power because SSI rates are typically low.^1^ Traditional SSI detection relies on manual chart reviews, which is time-consuming and prone to subjective error and facility-level variation. Therefore, it is important to utilize data available from the electronic medical record (EMR) to accurately identify SSIs and to decrease the burden of manual chart reviews.^2^

Semiautomated or fully automated algorithms are 2 strategies for using EMR data for SSI detection.^3^ A semiautomated algorithm classifies cases as high or low likelihood of a SSI after it has occurred based on EMR data (eg, antibiotic administration) and prioritizes the time-intensive manual review process based on algorithm output. This strategy aims to maintain high sensitivity to detect all cases of SSIs while decreasing the burden of chart reviews, and, therefore, it is suitable for surveillance. Prior studies have reported significant workload reduction by developing semiautomated SSI detection algorithms.^4^ Fully automated algorithms use EMR data to identify SSIs, without the added expense of time-intensive manual chart review. Due to the lack of confirmatory review, a fully automated algorithm aims to achieve a satisfactory positive predictive value (PPV) and specificity to identify SSIs, and it is suitable for research purposes.^3^

We aimed to develop a fully automated algorithm using data from the Veterans’ Affairs (VA) EMR to identify deep-incisional SSIs (ie, SSIs involving deep soft tissues of the incision, such as fascial and muscle layers) or organ-space SSIs (ie, SSIs involving any part of the body deeper than the fascial or muscle layers that was opened or manipulated during the operative procedure) after cardiac or orthopedic surgery.^5^

## Methods

We conducted a retrospective cohort study of patients who underwent 1 of 3 primary surgeries: coronary artery bypass grafting, valve replacement, or total joint arthroplasty (TJA, which indicates total hip arthroplasty or total knee arthroplasty) at 11 VA hospitals. The TJA cohort was derived from data collected between January 1, 2007, and March 31, 2018, and the cardiac surgery cohort was derived from data collected between January 1, 2010, and March 31, 2018. Data collection for the cardiac surgery cohort began in 2010 due to changes in the way the VA measured cardiac surgery outcomes before that date. Data from the VA’s integrated EMR were obtained from the Corporate Data Warehouse (CDW) through the Veterans’ Affairs Informatics and Computing Infrastructure (VINCI).

Data were collected for positive microbiology results, erythrocyte sedimentation rate (ESR), C-reactive protein (CRP), type and duration of inpatient antibiotic orders and outpatient oral antibiotic orders prescribed, length of stay at admission for primary surgery, readmission, consultation notes, and administrative code data (ACD) including *International Classification of Disease, Ninth or Tenth Revision* (ICD-9 or -10) codes for SSIs and current procedural terminology (CPT) codes for reoperation for an infection-related purpose. Consultation notes were extracted as text fields, and we conducted a search for SSI-related key words (Supplemental Table 1 online). Positive microbiology results were collected for blood cultures and local cultures and categorized as *Staphylococcus aureus*, coagulase-negative *Staphylococcus* spp, gram-positive cocci other than *Staphylococcus* spp, gram-negative rods, and other organisms. The highest value of CRP and ESR during follow-up period was recorded and used for analyses. ICD codes and CPT codes were selected to capture possible SSI diagnoses broadly, using codes described in previous studies.^6,7^

**Table 1.** Bivariate and Multivariate Analysis of Variables Associated With Surgical Site Infection

**A. Cardiac surgery** (N = 9,557)									
	No. (%) of Cases		Bivariate Analysis		Presence in 1,000 Bootstrap Replications, %	Multivariate Analysis			
Clinical characteristic	Mediastinitis or Endocarditis (n = 49)	No SSI(n = 9,508)	OR (95% CI)	*P* Value		OR (95% CI)	β-Coefficient (95% CI)	*P* Value	Point Score
Any consult note mentioning sternal or wound infection	12 (24.5)	27 (0.3)	3.9 (1.2–12.9)	.02	22.9				
ICD codes for mediastinitis	22 (44.9)	22 (0.2)	10.0 (3.7–26.6)	<.01	99.8	14.3 (5.6–36.0)	1.3	<.01	1
ICD codes for endocarditis	1 (2.0)	47 (0.5)	0.5 (<0.01–306.0)	.82	0				
ICD codes for other SSIs	44 (89.8)	519 (5.5)	17.2 (5.2–56.8)	<.01	99.4	42.7 (15.1–120.1)	1.9	<.01	2
ICD codes for other diagnosis for possible SSI	7 (14.3)	121 (1.3)	2.0 (0.6–6.0)	.24	11.3				
Sternal debridement	21 (42.9)	11 (0.1)	21.8 (6.9–69.0)	<.01	100	33.3 (11.7–94.6)	1.8	<.01	2
Reoperation	14 (28.6)	92 (1.0)	1.7 (0.6–4.8)	.32	77.2				
Readmission	30 (61.2)	1,632 (17.2)	1.5 (0.6–3.8)	.37	11.1				
Positive culture for MRSA	4 (8.2)	5 (0.1)	3.4 (0.6–19.5)	.17	84.4				
Positive culture for *Staphylococcus aureus*	13 (26.5)	37 (0.4)	5.5 (1.6–19.3)	<.01	93.3	7.3 (2.5–21.0)	1.0	<.01	1
Positive culture for GPC other than *Staphylococcus* spp	2 (4.1)	30 (0.3)	1.0 (0.1–17.2)	1.00	17.0				
Positive culture for GNR	8 (16.3)	98 (1.0)	0.8 (0.2–3.9)	.78	10.9				
Positive culture for other organisms	6 (12.2)	30 (0.3)	3.3 (0.6–18.3)	.18	15.3				
CRP >3 mg/dL	14 (28.6)	260 (2.7)	0.4 (0.1–1.8)	.26	74.7				
ESR >30 mm/h	13 (26.5)	258 (2.7)	4.2 (1.1–16.2)	.04	88.9				
ICD codes for respiratory infection	4 (8.2)	124 (1.3)	4.4 (0.9–21.0)	.06	88.1				
ICD codes for sepsis	8 (16.3)	100 (1.1)	2.1 (0.5–9.2)	.32	7.3				
Use of cefazolin, nafcillin or oxacillin	17 (34.7)	431 (4.5)	0.9 (0.3–2.6)	.90	6.0				
Any other antibiotic prescription	45 (91.8)	2,878 (30.3)	2.3 (0.6–8.6)	.23	12.2				
Length of primary hospital stay ≥14 d	22 (44.9)	1,840 (19.4)	2.1 (0.9–5.3)	.10	86.9				

**B. Total Joint Arthroplasty** (N = 12,992)									
Clinical Characteristic	No. (%) of Cases		Bivariate Analysis		Presence in 1,000 Bootstrap Replications, %	Multivariate Analysis			
	Deep-Incisional or Organ-Space SSI (n = 83)	No SSI (n = 12,909)	OR (95% CI)	*P* Value		OR (95% CI)	β-Coefficient (95% CI)	*P* Value	Point Score
Any consult note mentioning hip/knee/surgical infection	55 (66.3)	70 (0.5)	7.1 (3.0–16.6)	<.01	99.8	7.7 (3.4–17.1)	1.0	<.01	1
ICD codes for definite SSIs	53 (63.9)	74 (0.6)	17.9 (7.3–43.9)	<.01	100	27.7 (11.7–65.8)	1.7	<.01	2
ICD codes for other SSIs	27 (32.5)	99 (0.8)	0.8 (0.3–2.4)	.76	72.9				
ICD codes for other diagnosis for possible SSI	23 (27.7)	62 (0.5)	16.3 (5.6–47.5)	<.01	99.8	23.7 (8.7–64.4)	1.6	<.01	2
Reoperation	21 (25.3)	28 (0.2)	10.6 (3.6–31.1)	<.01	99.6	12.9 (4.6–35.9)	1.3	<.01	1
Readmission	60 (72.3)	1,725 (13.4)	2.7 (1.2–6.1)	.02	90.3	3.2 (1.5–6.9)	0.6	<.01	1
Positive culture for MRSA	15 (18.1)	41 (0.3)	0.8 (0.1–3.9)	.73	76.5				
Positive culture for *Staphylococcus aureus*	23 (27.7)	44 (0.3)	8.9 (2.7–29.4)	<.01	97.3	7.7 (2.8–21.0)	1.0	<.01	1
Positive culture for other *Staphylococcus* species	8 (9.6)	55 (0.4)	0.8 (0.2–3.3)	.76	12.2				
Positive culture for GPC other than *Staphylococcus* spp	7 (8.4)	11 (0.1)	4.0 (0.5–30.5)	.18	14.0				
Positive culture for GNR	9 (10.8)	35 (0.3)	1.9 (0.3–10.6)	.47	77.6				
Positive culture for other organisms	4 (4.8)	10 (0.1)	0.1 (0.01–0.6)	.01	89.1				
CRP >3 mg/dL	64 (77.1)	883 (6.8)	0.9 (0.3–3.3)	.92	4.3				
ESR >30 mm/h	61 (73.5)	682 (5.3)	1.6 (0.5–5.2)	.47	7.8				
ICD codes for endocarditis	0 (0)	1 (0.01)	<0.01 (<0.01–>999)	1.0	10.0				
ICD codes for respiratory infection	1 (1.2)	50 (0.4)	1.0 (0–40.3)	1.0	0				
ICD codes for sepsis	6 (7.2)	51 (0.4)	0.5 (0.1–4.8)	.59	7.6				
Use of vancomycin or piperacillin/tazobactam ≥ 6 days	34 (41.0)	116 (0.9)	7.9 (3.3–18.6)	<.01	99.6	7.8 (3.4–18.0)	1.0	<.01	1
Use of rifampin	24 (28.9)	13 (0.1)	6.1 (2.0–18.3)	<.01	95.5	5.8 (2.0–16.9)	0.9	<.01	1
Any other antibiotic prescription	73 (88.0)	2,452 (19.0)	4.1 (1.6–10.6)	<.01	29.3				
Length of primary hospital stay ≥ 14 days	7 (8.4)	270 (2.1)	3.8 (1.2–12.3)	.03	19.4				

Note. OR, odds ratio; CI, confidence interval; SSI, surgical site infection; ICD, *International Classification of Diseases*; MRSA, methicillin-resistant *Staphylococcus aureus*; ESR, erythrocyte sedimentation rate; CRP, C-reactive protein; GPC, gram-positive cocci; GNR, gram-negative rod.

The outcome of interest was mediastinitis, endocarditis, or deep-incisional or organ-space SSI within 30 days after surgery. The algorithm was tested for accuracy through comparison with outcomes collected via the Veterans’ Affairs Surgical Quality Improvement Program (VASQIP).^8^ VASQIP is a national program that uses a validated sampling algorithm to direct manual review for SSI surveillance, and it is very similar to the National Surgical Quality Improvement Program. VASQIP applies National Healthcare Safety Network (NHSN) definitions, with the exception of a 30-day end point for all types of SSI.^9^ However, VASQIP only reviews 70% of all major surgical cases. Patients without VASQIP review were excluded from the analysis. We elected to not to include superficial SSIs because superficial SSIs are subject to subjective bias and VASQIP did not collect superficial SSIs for large duration of study period.

We used multiple logistic regression analysis with repeated regular bootstrap procedure to select model variables. To build a model to predict SSIs, we started by assessing each candidate variable for bivariable associations with outcomes. We fit a multivariable logistic regression with SSIs as the outcome. To avoid overfitting of the model to the data, we selected variables using a backward elimination strategy to minimize Akaike’s information criterion (AIC). This variable selection was repeated 1,000 times using a bootstrapping method proposed by Austin and Tu^10^ to select only those variables identified as predictors in at least 90% of the bootstrap samples.

We then estimated coefficients of this multivariable logistic regression model by maximum likelihood estimation and used them to assign points for included variables. Coefficient values were approximated to the nearest integers for the ease of calculation and to improve practicality of the algorithm. We assigned points based on the created algorithm, and we measured sensitivity, specificity, PPV and negative predictive value (NPV) for each cutoff. We also used these summary statistics at each cutoff to create a receiver operating characteristic (ROC) curve to demonstrate the algorithm performance to predict SSIs. All statistical analyses were performed with SAS version 9.4 software (SAS Institution, Cary, NC).

The study was approved by the VA Central Institutional Review Board.

## Results

Among the 11 VA hospitals, we identified 13,341 cardiac surgeries and 18,914 TJAs during the study period. Of these, 9,557 (72.0%) cardiac surgeries and 12,992 (69.0%) TJAs were evaluated by VASQIP and were included in our final cohort. Moreover, 49 (0.5%) cardiac surgeries were classified mediastinitis or endocarditis in VASQIP, and 83 TJAs (0.6%) were classified as deep-incisional or organ-space SSIs.

**Fig. 1.**
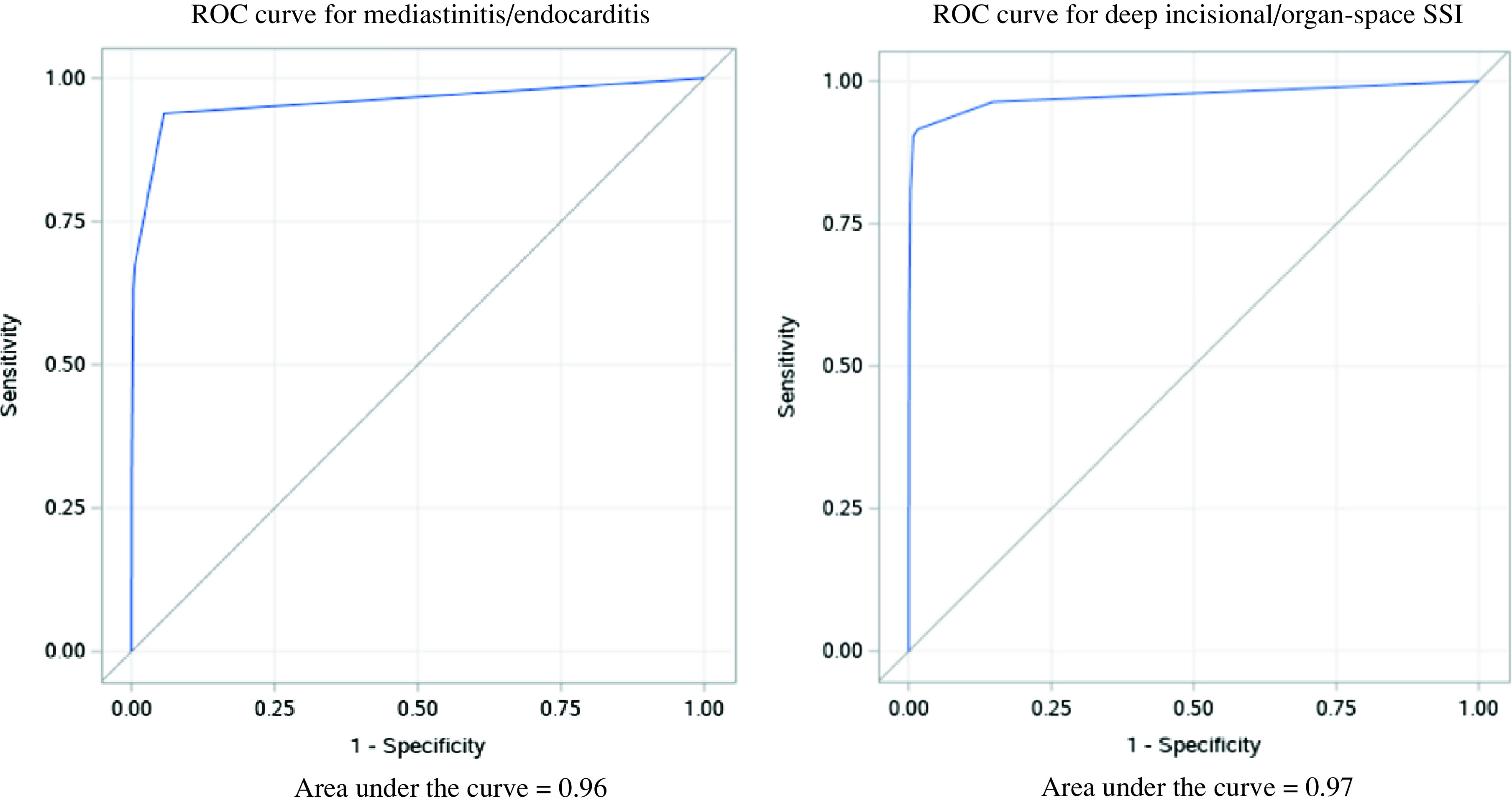
Receiver operating characteristic (ROC) curve for surgical site infection (SSI) detection algorithms.

The ICD codes for infection, subsequent surgery (sternal debridement in cardiac surgeries and reoperation in TJAs) and positive cultures for *S. aureus* were included in both models (Table 1). Sensitivities, specificities, PPVs and NPVs in varying cutoffs are shown in Table 2. With ≥60% sensitivity, the PPV of the SSI detection algorithm after cardiac surgeries was 52.5%. With 80% sensitivity, the PPV of the SSI detection algorithm after TJA was 62.0%. The area under the curve (AUC) of the receiver operating characteristic (ROC) curve for SSI detection algorithms after cardiac surgeries and TJAs were 0.96 and 0.97 in the final models, respectively (Fig. 1).

**Table 2.** Sensitivities, Specificities, PPV Positive Predictive Values and NPV Negative Predictive Values of Surgical Site Infection Detection Algorithms, With Varying Cutoffs

Cutoff	Cases Detected as Having SSIs, No. (%)	Sensitivity, %	Specificity, %	Positive Predictive Value, %	Negative Predictive Value, %
**Cardiac surgery** (N = 9,557)^a^					
1	581 (6.1)	93.9	94.4	7.9	99.9
2	86 (0.9)	67.4	99.4	38.4	99.8
3	59 (0.6)	63.3	99.7	52.5	99.8
4	20 (0.2)	32.7	99.9	80.0	99.7
5	14 (0.2)	24.5	99.9	85.7	99.6
6	5 (0.1)	10.2	100	100	99.5
**Total joint arthroplasty** (N = 12,992)^b^					
1	1,983 (15.3)	96.4	85.3	4.0	99.9
2	277 (2.1)	91.6	98.4	27.4	99.9
3	178 (1.4)	90.4	99.2	42.1	99.9
4	108 (0.8)	80.7	99.7	62.0	99.9
5	63 (0.5)	59.0	99.9	77.8	99.7
6	21 (0.2)	20.5	99.9	81.0	99.5
7	6 (0.1)	6.0	99.9	83.3	99.4

Note. PPV, positive predictive values; NPV, negative predictive values; SSI, surgical site infection; ESR, erythrocyte sedimentation rate; ICD, *International Classification of Diseases.*

a Positive culture for *Staphylococcus aureus* = 1 points, sternal debridement = 2 points, ICD codes for other SSIs = 2 points, ICD codes for mediastinitis = 1 point.

b ICD codes for definite SSIs = 2 points, ICD codes for other diagnosis for possible SSIs = 2 points, any consultation note mentioning hip or knee surgical infection = 1 point, reoperation = 1 point, readmission = 1 point, positive culture for *Staphylococcus aureus* = 1 point, use of vancomycin or piperacillin/tazobactam ≥6 days = 1 point, use of rifampin = 1 point.

## Discussion

We used data from the existing VA EMR to develop a fully automated SSI detection algorithms for cardiac surgeries and TJAs. With VASQIP as a reference standard, the algorithms achieved high specificities and NPVs, with PPVs of 52.5% for cardiac surgeries and 62.0% for TJAs while maintaining at least 60% sensitivity. Considering the low prevalence rate of SSIs, our algorithms were successful in identifying a majority of patients with a true SSI while simultaneously reducing false-positive cases.

Our study utilized detailed clinical data combined with ACD to differentiate cases with and without SSIs. ACD are widely used in surveillance and public reporting programs but are insufficient as a sole indicator for detecting SSIs.^11^ In addition to ACD, positive clinical cultures for *S. aureus* were assigned high points. The possible rationale behind this is that *S. aureus* is the most common pathogen causing postoperative mediastinitis and early-onset prosthetic joint infections. In our study, the performance of the SSI detection algorithm for TJAs was better than that for cardiac surgeries. This difference is probably due to the better performance of ACD used for SSIs after orthopedic surgeries, compared to those used for SSIs after cardiac surgeries.^11,12^

This study has several limitations. First, we only evaluated patients for 30 days after index surgery because VASQIP only detects SSIs within 30 days. This method differs from NHSN surveillance that detects SSIs within 90 days for TJA and cardiac surgeries. We might have missed SSIs caused by more indolent organisms with onset beyond the 30-day period. Second, only deep-incisional and organ-space SSIs were targeted in our algorithm because the symptoms and definitions associated with superficial infections are vague. Third, different ACD (ICD-9/-10) were used in different time frames during study period. ICD-9/-10 translations do not always align and produce discontinuity in some diagnoses.^13^ Fourth, we evaluated only positive clinical cultures; thus, we might have missed some culture-negative SSIs. However, this limitation was mitigated by other elements of the algorithm, which would have flagged patients in other ways, such as ACD or antimicrobial orders. Finally, due to limitations of the EMR, we could not obtain outpatient intravenous antibiotic data; however, the vast majority of patients with deep-incisional or organ-space SSIs are first evaluated on an inpatient basis; thus, those orders would have been included in the algorithm.

Future research to improve these algorithms should include machine-learning approaches or advanced key word searches for infection-related terms, as has been applied for measurement of infections following cardiac device procedures within the VA.^14^ These algorithms should also be validated in other healthcare systems with EMR data using NHSN data as the gold standard.

Current research studies of interventions to prevent SSI rely on either ACD alone or on data from intensive chart review to assess whether the interventions were successful.^11,15^ These algorithms will improve our ability to accurately evaluate SSI interventions in large healthcare systems. In the VA context, these algorithms can be combined with VASQIP data to assess SSIs among all relevant surgical patients in the VA system, rather than only the proportion of patients assessed by VASQIP.

## Supplementary material

For supplementary material accompanying this paper visit https://doi.org/10.1017/ice.2020.1387. click here to view supplementary material
