# Supplementary material for: Development of a fully automated surgical site infection detection algorithm for use in cardiac and orthopedic surgery research
Source: Infect Control Hosp Epidemiol. 2021 Feb 23;42(10):1215–20. doi: 10.1017/ice.2020.1387 (PMC8506349; doi:10.1017/ice.2020.1387)
Supplement: Supplementary file 1 [file S0899823X20013872sup.zip › S0899823X20013872sup001.docx]

Supplemental Table 1. Description of variables used in the SSI detection algorithm

| Variables | Description |
| --- | --- |
| Cardiac surgeries | |
| Any consult note mentioning sternal or wound infection (30 days post-surgery) | Provisional diagnoses with ‘STERN’ or ‘WOUND’ or ‘SURGICAL’ or ‘POSTOPERATIVE’ or “CHEST’ in combination with ‘INFEC’  Exclude diagnoses with ‘POSSIBLE’, ‘SUPERFICIAL’, ‘GROIN’, ‘ANKLE’, ‘BACK’, ‘SOFT’, ‘CELLULITIS’, ‘NO INFECT’, ‘PROBA’, ‘EVAL’, ‘QUESTION’, ‘SUSPEC’ |
| ICD codes for mediastinitis (45 days post-surgery) | ICD-9: ‘519.2’  ICD-10: ‘J98.5’, ‘J98.51’ |
| ICD codes for endocarditis (30 days post-surgery) | ICD-9: ‘421.9’, ‘424.91’, ‘424.99’  ICD-10: ‘I01.1’, ‘I33.0’, ‘I33.9’ |
| ICD codes for other SSIs (45 days post-surgery) | ICD-9: ‘998.51’, ‘998.59’  ICD-10: ‘T81.31XA’, ‘T81.32XA’, ‘T81.4XXA’ |
| ICD codes for other diagnosis for possible SSI (30 days post-surgery) | ICD-9: ‘682.2’, ‘730.08’, ‘730.28’, ‘996.61’  ICD-10: ‘J85.3’, ‘L03.319’, ‘M46.20’, ‘T81.89XA’, ‘T82.6XXA’ |
| Sternal debridement (30 days post-surgery) | CPT code: ‘21627’ |
| Positive culture for MRSA (45 days post-surgery) | MRSA grew from clinical culture from the following body sites: wound, sternum, sternal angle, sternal, skin of chest, mediastinum, chest wall, chest, bone, blood, abscess material |
| Re-operation (30 days post-surgery) | ICD-9: ‘86.05’, ‘86.22’, ‘86.3’, ‘86.4’  ICD-10: ‘0HB5XZZ’, ‘0HBHXZZ’, ‘0HBJXZZ’, ‘0HBKXZZ’, ‘0HBLXZZ’, ‘0JB63ZZ’, ‘0JBL3ZZ’, ‘0JBM3ZZ’, ‘0JBN3ZZ’, ‘0JBP3ZZ’, ‘0JB60ZZ’, ‘0JBL0ZZ’, ‘0JBM0ZZ’, ‘0JBN0ZZ’, ‘0JBP0ZZ’, ‘0YB90ZZ’, ‘0YB93ZZ’, ‘0YB94ZZ’, ‘0YBB0ZZ’, ‘0YBB3ZZ’, ‘0YBB4ZZ’, ‘0YBC0ZZ’, ‘0YBC3ZZ’, ‘0YBC4ZZ’, ‘0YBD0ZZ’, ‘0YBD3ZZ’, ‘0YBD4ZZ’, ‘0YBF0ZZ’, ‘0YBF3ZZ’, ‘0YBF4ZZ’, ‘0YBG0ZZ’, ‘0YBG3ZZ’, ‘0YBG4ZZ’, ‘0YBH0ZZ’, ‘0YBH3ZZ’, ‘0YBH4ZZ’, ‘0YBJ0ZZ’, ‘0YBJ3ZZ’, ‘0YBJ4ZZ’, ‘0HPPX7Z’, ‘0JC60ZZ’, ‘0JCL0ZZ’, ‘0JCM0ZZ’, ‘0JCN0ZZ’, ‘0JCP0ZZ’, ‘0YP90YZ’, ‘0YPB0YZ’, ‘0HB5XZZ’, ‘0HBHXZZ’, ‘0HBJXZZ’, ‘0HBKXZZ’, ‘0HBLXZZ’, ‘0JB60ZZ’, ‘0JBL0ZZ’, ‘0JBM0ZZ’, ‘0JBN0ZZ’, ‘0JBP0ZZ’  CPT code: ‘11000’, ‘11001’, ‘11005’, ‘11006’, ‘11008’, ‘11010’, ‘11011’, ‘11012’, ‘11042’, ‘11043’, ‘11044’, ‘11045’, ‘11046’, ‘11047’, ‘15000’, ‘15001’, ‘20680’, ‘21620’, ‘21630’, ‘21670’, ‘21705’, ‘21750’, ‘97597’, ‘97598’ |
| Positive culture for *Staphylococcus aureus* (45 days post-surgery) | *Staphylococcus aureus* grew from clinical culture from the following body sites: wound, sternum, sternal angle, sternal, skin of chest, mediastinum, chest wall, chest, bone, blood, abscess material |
| Positive culture for GPC other than *Staphylococcus* spp. (45 days post-surgery) | GPC other than *Staphylococcus* spp. grew from clinical culture from the following body sites: wound, sternum, sternal angle, sternal, skin of chest, mediastinum, chest wall, chest, bone, blood, abscess material |
| Positive culture for GNR (45 days post-surgery) | GNR grew from clinical culture from the following body sites: wound, sternum, sternal angle, sternal, skin of chest, mediastinum, chest wall, chest, bone, blood, abscess material |
| Positive culture for other organisms (45 days post-surgery) | Other organisms grew from clinical culture from the following body sites: wound, sternum, sternal angle, sternal, skin of chest, mediastinum, chest wall, chest, bone, blood, abscess material |
| CRP >3mg/dL | Any point during follow-up |
| ESR >30 mm/h | Any point during follow-up |
| Any other antibiotic prescription (3-30 days post-surgery) | Antibiotic prescription other than nafcillin, oxacillin, cefazolin |
| ICD codes for respiratory infection (30 days post-surgery) | ICD-9: ‘482.41’, ‘482.9’, ‘510.9’, ‘511.1’  ICD-10: ‘J06.9’, ‘J15.211’, ‘J15.9’, ‘J18.9’, ‘J86.9’ |
| ICD codes for sepsis (30 days post-surgery) | ICD-9: ‘995.91’, ‘995.92’  ICD-10: ‘A41.9’, ‘R65.20’, ‘R65.21’ |
| Total joint arthroplasties | |
| Any consult note mentioning hip/knee/surgical infection (30 days post-surgery) | Provisional diagnoses with ‘WOUND’ or ‘SURGICAL’ or ‘POSTOPERATIVE’ or ‘TKR’ or ‘PROSTHE’ or ‘KNEE’ or ‘HIP’ or ‘THA’ or ‘ARTHRITIS’ or ‘JOINT’ or ‘LEG’ or ‘ARTHRO’ in combination with ‘INFE’  Exclude diagnoses with ‘POSSIB’, ‘SUPERFICIAL’, GROIN’, ‘INITIAL’, ANKLE’, ‘BACK’, ‘SOFT’, ‘CELLULITIS’, ‘NO INFECT’, ‘PROBA’, EVAL’, ‘QUESTION’, ‘SUSPEC’, ‘RESPIRA’, ‘?’, ‘ARTHRITIS’, ‘S/P’, ‘TRAVEL’, ‘ADMISSION’, ‘DENTAL’ |
| ICD codes for definite SSIs (30 days post-surgery) | ICD-9: ‘711.05’, ‘711.06’, ‘711.95’, ‘711.96’, ‘730.05’, ‘730.06’, ‘730.08’, ‘730.25’, ‘730.26’, ‘730.28’, ‘730.29’, ‘730.96’, ‘730.98’, ‘996.66’, ‘996.67’, ‘996.69’  ICD-10: ‘M00.051’, ‘M00.061’, ‘M00.062’, ‘M00.851’, ‘M00.852’, ‘M00.859’, ‘M00.861’, ‘M00.862’, ‘M00.869’, ‘M46.18’, ‘M46.20’, ‘M86.18’, ‘M86.28’ |
| ICD codes for other SSIs (30 days post-surgery) | ICD-9: ‘998.51’, ‘998.59’  ICD-10: ‘T81.31XA’, ‘T81.32XA’, ‘T81.4XXA’ |
| ICD codes for other diagnosis for possible SSI (30 days post-surgery) | ICD-9: ‘996.44’, ‘996.45’, ‘996.46’, ‘996.47’, ‘996.49’, ‘996.77’, ‘996.78’, ‘997.62’  ICD-10: ‘T84.50XA’, ‘T84.50XD’, ‘T84.50XS’, ‘T84.51XA’, ‘T84.51XD’, ‘T84.51XS’, ‘T84.52XA’, ‘T84.52XD’, ‘T84.52XS’, ‘T84.53XA’, ‘T84.53XD’, ‘T84.53XS’, ‘T84.54XA’, ‘T84.54XD’, ‘T84.54XS’, ‘T84.59XA’, ‘T84.59XD’, ‘T84.59XS’, ‘T84.7XXA’, ‘T84.7XXD’, ‘T84.7XXS’ |
| Positive culture for *Staphylococcus aureus* (45 days post-surgery) | *Staphylococcus aureus* grew from clinical culture from the following body sites: abscess, abscess material, acetabulum, blood, body fluid, bone, bursa, calf of leg, femoral, femoral head, femur, fine needle aspirate, fluid, hip, hip joint, joint, knee, knee joint, lower extremity, thigh, patella, skin of knee, suture line of skin, synovial fluid, synovial joint, synovial tissue, synovium, wound |
| Positive culture for MRSA (45 days post-surgery) | MRSA grew from clinical culture from the following body sites: abscess, abscess material, acetabulum, blood, body fluid, bone, bursa, calf of leg, femoral, femoral head, femur, fine needle aspirate, fluid, hip, hip joint, joint, knee, knee joint, lower extremity, thigh, patella, skin of knee, suture line of skin, synovial fluid, synovial joint, synovial tissue, synovium, wound |
| Positive culture for other *Staphylococcus* species (45 days post-surgery) | *Staphylococcus* species other than *Staphylococcus aureus* grew from clinical culture from the following body sites: abscess, abscess material, acetabulum, blood, body fluid, bone, bursa, calf of leg, femoral, femoral head, femur, fine needle aspirate, fluid, hip, hip joint, joint, knee, knee joint, lower extremity, thigh, patella, skin of knee, suture line of skin, synovial fluid, synovial joint, synovial tissue, synovium, wound |
| Positive culture for GPC other than *Staphylococcus* spp. (45 days post-surgery) | GPC other than *Staphylococcus* spp. grew from clinical culture from the following body sites: abscess, abscess material, acetabulum, blood, body fluid, bone, bursa, calf of leg, femoral, femoral head, femur, fine needle aspirate, fluid, hip, hip joint, joint, knee, knee joint, lower extremity, thigh, patella, skin of knee, suture line of skin, synovial fluid, synovial joint, synovial tissue, synovium, wound |
| Positive culture for GNR (45 days post-surgery) | GNR grew from clinical culture from the following body sites: abscess, abscess material, acetabulum, blood, body fluid, bone, bursa, calf of leg, femoral, femoral head, femur, fine needle aspirate, fluid, hip, hip joint, joint, knee, knee joint, lower extremity, thigh, patella, skin of knee, suture line of skin, synovial fluid, synovial joint, synovial tissue, synovium, wound |
| Positive culture for other organisms (45 days post-surgery) | Other organisms grew from clinical culture from the following body sites: abscess, abscess material, acetabulum, blood, body fluid, bone, bursa, calf of leg, femoral, femoral head, femur, fine needle aspirate, fluid, hip, hip joint, joint, knee, knee joint, lower extremity, thigh, patella, skin of knee, suture line of skin, synovial fluid, synovial joint, synovial tissue, synovium, wound |
| Re-operation (30 days post-surgery) | ICD-9: ‘80.15’, ‘80.16’, ‘80.36’, ‘80.75’, ‘81.55’, ‘84.56’  ICD-10: ‘0SWC0JZ’, ‘0SWC3JZ’, ‘0SWC4JZ’, ‘0SWD0JZ’, ‘0SWD3JZ’, ‘0SWD4JZ’, ‘0S9B0ZZ’, ‘0S9B0ZX’, ‘0S9B3ZZ’, ‘0S9B3ZX’, ‘0S9D0ZZ’, ‘0S9D0ZX’, ‘0S9D3ZZ’, ‘0S9D3ZX’, ‘0S990ZZ’, ‘0S990ZX’, ‘0S993ZZ’, ‘0S993ZX’, ‘0S9C0ZZ’, ‘0S9C0ZX’, ‘0S9C3ZZ, ‘0S9C3ZX’, ‘0SBB0ZZ’, ‘0SBB0ZX’, ‘0SBD0ZZ’, ‘0SBD0ZX’, ‘0SB90ZZ’, ‘0SB90ZX’, ‘0SB93ZZ’, ‘0SB93ZX’, ‘0SBC0ZZ’, ‘0SBC0ZX’, ‘0SCB0ZZ’, ‘0SCD0ZZ’, ‘0SC90ZZ’, ‘0SCC0ZZ’, ‘0SHB04Z’, ‘0SHD04Z’, ‘0SH904Z’, ‘0SHC04Z’, ‘0SHB08Z’, ‘0SHD08Z, ‘0SH908Z’, ‘0SHC08Z’, ‘0SPBX4Z’, ‘0SPB04Z’, ‘0SPDX4Z’, ‘0SPD04Z’, ‘0SP9X4Z’, ‘0SP904Z’, ‘0SPCX4Z’, ‘0SPC04Z’, ‘0SPB09Z’, ‘0SPD09Z’, ‘0SP909Z’, ‘0SPC09Z’, ‘0SPB08Z’, ‘0SPD08Z’, ‘0SP908Z’, ‘0SPC08Z’, ‘0STB0ZZ’, ‘0STD0ZZ’, ‘0ST90ZZ’, ‘0STC0ZZ’, ‘0SWBX4Z’, ‘0SWB04Z’, ‘0SWDX4Z’, ‘0SWD04Z’, ‘0SW9X4Z’, ‘0SW904Z’, ‘0SWCX4Z’, ‘0SWC04Z’, ‘0SWB09Z’, ‘0SWD09Z’, ‘0SW909Z’, ‘0SWC09Z’, ‘0SWBX8Z’, ‘0SWB08Z’, ‘0SWDX8Z’, ‘0SWD08Z’, ‘0SW9X8Z’, ‘0SW908Z’, ‘0SWCX8Z’, ‘0SWC08Z’, ‘0SBB3ZX’, ‘0SBB3ZZ’, ‘0SBC3ZX’, ‘0SBC3ZZ’, ‘0SBD3ZX’, ‘0SBD3ZZ’, ‘0SC93ZZ’, ‘0SCB3ZZ’, ‘0SCC3ZZ’, ‘0SCD3ZZ’, ‘0SH934Z’, ‘0SH938Z’, ‘0SHB34Z’, ‘0SHB38Z’, ‘0SHC34Z’, ‘0SHC38Z’, ‘0SHD34Z’, ‘0SHD38Z’, ‘0SP934Z’, ‘0SP938Z’, ‘0SPB34Z’, ‘0SPB38Z’, ‘0SPC34Z’, ‘0SPC38Z’, ‘0SPD34Z’, ‘0SPD38Z’, ‘0SW934Z’, ‘0SW938Z’, ‘0SWB34Z’, ‘0SWB38Z’, ‘0SWC34Z’, ‘0SWC38Z’, ‘0SWD34Z’, ‘0SWD38Z’, ‘0S9C4ZX’, ‘0S9C4ZZ, ‘0S9D4ZX’, ‘0S9D4ZZ’, ‘0S994ZX’, ‘0S994ZZ’, ‘0S9B4ZX’, ‘0S9B4ZZ’, ‘0SB94ZX’, ‘0SB94ZZ’, ‘0SBB4ZX’, ‘0SBB4ZZ’, ‘0SBC4ZX’, ‘0SBC4ZZ’, ‘0SBD4ZX’, ‘0SBD4ZZ’, ‘0SC94ZZ’, ‘0SCB4ZZ’, ‘0SCC4ZZ’, ‘0SCD4ZZ’, ‘0SH944Z’, ‘0SH948Z’, ‘0SHB44Z’, ‘0SHB48Z’, ‘0SHC44Z’, ‘0SHC48Z’, ‘0SHD44Z’, ‘0SHD48Z’, ‘0SP944Z’, ‘0SP948Z’, ‘0SPB44Z’, ‘0SPB48Z’, ‘0SPC44Z’, ‘0SPC48Z’, ‘0SPD44Z’, ‘0SPD48Z’, ‘0SW944Z’, ‘0SW948Z’, ‘0SWB44Z’, ‘0SWB48Z’, ‘0SWC44Z’, ‘0SWC48Z’, ‘0SWD44Z’, ‘0SWD48Z’  CPT code: ‘21871’, ‘27030’, ‘27310’ |
| ESR >30 mm/h | Any point during follow-up |
| CRP >3mg/dL | Any point during follow-up |
| Any other antibiotic prescription (3-30 days post-surgery) | Antibiotic prescription other than rifampin, >= 6 days of vancomycin or >= 6 days of piperacillin/tazobactam |
| ICD codes for respiratory infection (30 days post-surgery) | ICD-9: ‘482.41’, ‘482.9’, ‘510.9’, ‘511.1’  ICD-10: ‘J06.9’, ‘J15.211’, ‘J15.9’, ‘J18.9’, ‘J86.9’ |
| ICD codes for sepsis (30 days post-surgery) | ICD-9: ‘995.91’, ‘995.92’  ICD-10: ‘A41.9’, ‘R65.20’, ‘R65.21’ |
| ICD codes for endocarditis (30 days post-surgery) | ICD-9: ‘421.9’, ‘424.91’, ‘424.99’  ICD-10: ‘I01.1’, ‘I33.0’, ‘I33.9’ |

SSI: Surgical site infection, ICD: International Classification of Diseases, MRSA: Methicillin-resistant *Staphylococcus aureus*, ESR: Erythrocyte sedimentation rate, CRP: C-reactive protein, CPT: Current Procedural Terminology, GPC: Gram-positive cocci, GNR: Gram-negative rod
