# Supplementary material for: Development of a fully automated surgical site infection detection algorithm for use in cardiac and orthopedic surgery research
Source: Infect Control Hosp Epidemiol. 2021 Feb 23;42(10):1215–20. doi: 10.1017/ice.2020.1387 (PMC8506349; doi:10.1017/ice.2020.1387)
Supplement: Supplementary file 1 [file S0899823X20013872sup.zip › S0899823X20013872sup002.pptx]

## Slide 1
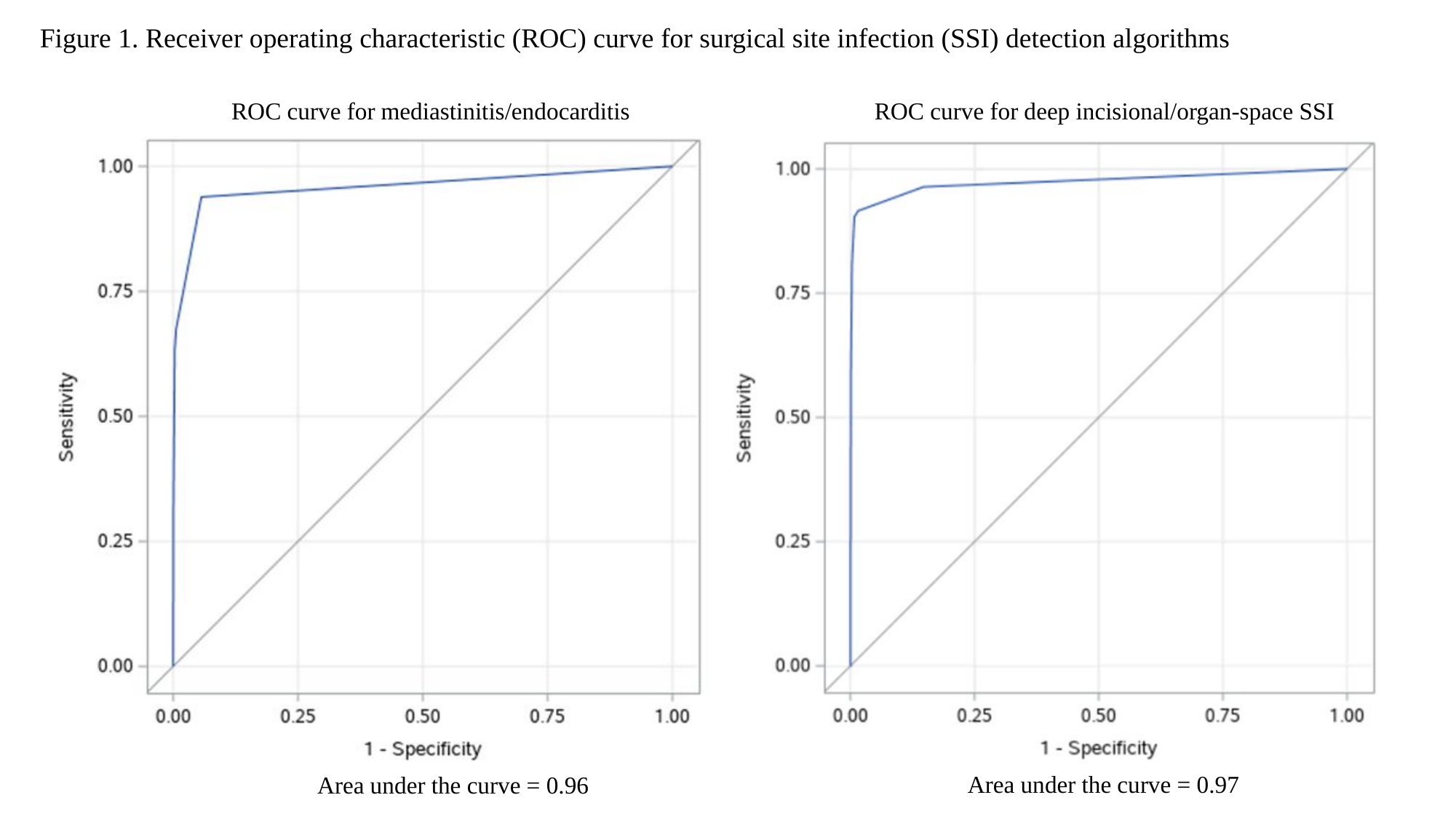

Figure 1. Receiver operating characteristic (ROC) curve for surgical site infection (SSI) detection algorithms
ROC curve for mediastinitis/endocarditis
ROC curve for deep incisional/organ-space SSI
Area under the curve = 0.97
Area under the curve = 0.96
